# Psychological Distress Among Parents of Children With Chronic Health Conditions and Its Association With Unmet Supportive Care Needs and Children’s Quality of Life

**DOI:** 10.1093/jpepsy/jsad074

**Published:** 2023-10-14

**Authors:** Sangeetha Thomas, Nicholas P Ryan, Linda K Byrne, Christel Hendrieckx, Victoria White

**Affiliations:** Faculty of Health, School of Psychology, Deakin University, Australia; Faculty of Health, School of Psychology, Deakin University, Australia; Department of Paediatrics, University of Melbourne, Australia; Faculty of Health, School of Psychology, Deakin University, Australia; Faculty of Psychology, Counselling and Psychotherapy, The Cairnmillar Institute, Australia; Faculty of Health, School of Psychology, Deakin University, Australia; The Australian Centre for Behavioural Research in Diabetes, Australia; Faculty of Health, Institute of Health Transformation, Deakin University, Australia; Faculty of Health, School of Psychology, Deakin University, Australia; Centre for Behavioural Research in Cancer, Cancer Council Victoria, Melbourne, Australia

**Keywords:** anxiety, chronic illness, depression, pediatrics, quality of life, stress, unmet needs, unmet supportive care needs

## Abstract

**Objective:**

To assess parent psychological distress in families of children with common chronic health conditions (CHC) and to explore relationships between parent psychological distress, unmet supportive care needs and children’s quality of life (QoL).

**Method:**

Cross-sectional study involving parents of children diagnosed with a common CHC between 0 and 12 years of age and who had received treatment within the last 5 years. Eligible parents completed an online survey, that included the Depression Anxiety Stress Scale (DASS-21) assessing distress in parents and a 34-item assessment of unmet supportive care needs across 6 domains. Parents completed ratings of their child’s current functioning (QoL) using the 23-item PedsQL. Multivariable regression models examined the relative association between unmet needs, children’s QoL and parents’ depression, anxiety, and stress.

**Results:**

The sample consisted of 194 parents of children with congenital heart disease (*n*=97; 50%), diabetes (*n*=50; 26%), cancer (*n*=39; 20%), and asthma (*n*=8; 4%). A significant proportion of parents had moderate–severe symptoms of depression (26%), anxiety (38%), and stress (40%). Of the PedsQL scales, the poorest outcomes were found for emotional and school functioning. Multivariable analyses showed that both higher unmet needs and poorer child emotional functioning were associated with parent depression, anxiety, and stress symptoms.

**Conclusion:**

Evidence linking parent distress symptoms to higher unmet needs and poorer child emotional functioning suggests these factors may be targets for interventions to alleviate parent distress. Longitudinal research using larger samples is required to replicate findings, and clarify the magnitude and direction of associations.

## Introduction

Chronic Health Conditions (CHCs) are health problems that require ongoing clinical review for over 12 months and may involve ongoing specialist medical care and hospital admissions (Wijlaars et al., 2016). Although children can be affected by a vast number of CHCs, recent evidence suggests that asthma, congenital heart disease (CHD), type 1 diabetes (T1D), and cancer are among the most commonly treated physical health conditions among children in Australia and the United States of America (AIHW, 2022; Miller et al., 2016; Van Cleave, 2010).

CHCs in childhood can significantly interfere with aspects of children’s physical, social, emotional, and academic development, which may, in turn, contribute to impairments in child quality of life (QoL) and family well-being (ABS, 2022; AIHW, 2022; Miller et al., 2016; Van Cleave, 2010). While initial diagnosis and treatment of childhood CHCs have been linked to a range of psychosocial challenges for affected children and their families, relatively few studies have evaluated the long-term psychological impacts of CHCs on families (Pardini, 2008). Moreover, the design and implementation of psychosocial interventions for these families are hindered by a dearth of knowledge regarding the health, social, financial, and educational support needs of children with common CHCs (ABS, 2022; Miller et al., 2016; Van Cleave, 2010).

### Impact of Pediatric Chronic Illness on Parent and Child Psychological Health

Parents or primary caregivers inevitably play a critical role in managing the various medical and psychosocial impacts of their child’s CHC (Kish et al., 2018). For example, this might include managing medical appointments, implementing and monitoring medication regimes (e.g., insulin injections, appropriate use of inhalers) (Javalkar et al., 2017). When these increased responsibilities are coupled with the additional demands of managing the impact of illness on their child’s emotional, social, and physical health and well-being, it is not surprising that many parents of children with chronic illness experience elevated stress, anxiety, and depression across the disease trajectory (Chen et al., 2023; Cousino & Hazen, 2013; Jantien Vrijmoet-Wiersma et al., 2008; Kolaitis et al., 2017; Whittemore et al., 2012). This situation is highlighted in a systematic review of mental health outcomes in parents of children with T1D, which found that approximately 34% of parents experience anxiety/depression at diagnosis and that 19% of parents experience elevated distress up to four years post-diagnosis (Whittemore et al., 2012). Overall, while increasing evidence suggests that parents of children with CHCs are at elevated risk for persisting psychological distress (Chen et al., 2023; Cousino & Hazen, 2013; Jantien Vrijmoet-Wiersma et al., 2008; Kolaitis et al., 2017; Whittemore et al., 2012), the specific factors associated with this heightened psychological vulnerability in parents are poorly understood.

Parent psychological distress in families of children with CHCs is likely associated with a range of factors, including family demographic variables, reduced child QoL, and higher levels of treatment burden (Bakula et al., 2020; Saßmann et al., 2022). For example, the results of a meta-analysis involving 14 studies (12 cross-sectional) in child cancer populations found significant associations between parent distress and poorer child functioning on measures of children’s QoL (Bakula et al., 2020). While ecological models of child development (Bronfenbrenner, 1979; Kazak, 1989) posit that parent and child functioning have reciprocal, bidirectional influences on one another, existing research in childhood chronic illness populations has mostly examined the presence of unidirectional associations, such that parent-related characteristics are hypothesized to strengthen and/or limit children’s functioning and QoL (Pardini, 2008; Robinson et al., 2016; Yan & Ansari, 2016).

Although the presence of a unidirectional association between parent and child functioning is unlikely to reflect the complex clinical reality of childhood chronic illnesses, studies in this area have seldom explored whether lower child functioning and poorer child QoL may contribute to greater parental distress in families of children with CHCs. Given that lower child functioning and poorer child QoL are likely to lead to additional caregiving responsibilities (Leeman et al., 2016; Smith et al., 2022), increased caregiver emotional burden (Flury et al., 2011; Robinson et al., 2016; Whittemore et al., 2012), and reduced opportunities for self-care (Aziza et al., 2019; Robinson et al., 2016; Saßmann et al., 2022), further research is needed to evaluate whether poorer child functioning and QoL is a risk factor associated with psychological distress among parents of children with CHCs.

### Unmet Supportive Care Needs

Parent psychological distress is also likely associated with *supportive care needs* (SCN), which refer to the information, resources, and/or support parents and children require across the course of the CHC trajectory (Kerr et al., 2004). Accordingly, *unmet* needs refer to gaps between the support available through standard care pathways and the support that families require to navigate the impact of the illness on various aspects of the child’s and the family’s life. Drawing on studies of SCN in adults living with cancer, Fitch (2008) proposed the SCN framework, which groups SCN into physical, emotional, social, psychological, informational, spiritual, health care, and practical domains (Fitch, 2008). While originally developed for adults with cancer, the framework was subsequently been extended to conceptualize the experiences and needs of caregivers, including carers of adults and children living with cancer (Girgis et al., 2011; Kerr et al., 2004), as well as carers of children with life-limiting, rare, and other CHCs (Denham et al., 2020; Gill et al., 2021; Pelentsov et al., 2015).

In the substantial body of work examining unmet needs in adults with cancer and their carers, unmet SCN (USCN) are consistently associated with greater levels of psychological distress, including depression, anxiety, and stress (Girgis et al., 2011; Lambert et al., 2012; Naser et al., 2021; Niedzwiedz et al., 2019) and lower levels of QoL (Jang & Jeong, 2021; Lisy et al., 2019; Wang et al., 2018). Other work in carers of adult cancer patients has shown that carer QoL and psychological distress are related to the level of unmet needs experienced by both the carer themselves and the individual with cancer (Jang & Jeong, 2021; Kim & Carver, 2019; Wang et al., 2021). These findings may have relevance for understanding the potential role of unmet needs in contributing to psychological distress and functioning in families of children with CHCs; however, few studies have examined these associations in this population. A study of parents of children with cancer (*N* = 100) looked at the association between anxiety and depression and unmet needs and found that unmet needs were higher in parents with clinical levels of anxiety but were not associated with clinical levels of depression (Aziza et al., 2019). In another study involving 286 parents of children with cancer, higher parental depression symptoms mediated the association between the level of unmet needs reported by parents and children’s QoL (Wang et al., 2022).

Though research involving families of children with T1D, CHD, and asthma has helped to characterize the unmet needs of these families, there has been limited exploration of the extent to which USCN may relate to the severity of parent psychological distress (Collier et al., 2001; Levert et al., 2017). Instead, research in this area has typically studied the relationship between socio-demographic factors and the daily, emotional, and physical caregiving burdens experienced by parents of children with chronic illness (Saßmann et al., 2022). For example, one recent study involving 1,107 parents of children with T1D found that levels of parent emotional burden were associated with the parent’s gender (mother), with high levels of daily and physical burdens linked to parent age and single-parent family status (Saßmann et al., 2022). However, this research did not examine whether greater levels of parental distress and/or emotional burden were linked to high levels of USCN and/or aspects of child functioning, including lower child QoL.

To address substantial gaps in knowledge, this exploratory, cross-sectional investigation aims to characterize the nature and frequency of psychological distress in parents of children with common CHCs and to explore factors associated with parent psychological distress in these high-risk families. Specifically, the study aimed to (1) assess the frequency and severity of anxiety, depression, and stress symptoms in parents of children with a medically confirmed CHC and (2) explore the association between parental distress, unmet needs, and child’s QoL.

## Method

### Study Design and Setting

Through convenience sampling, we invited parents of children with common CHCs to complete an online, cross-sectional survey. This is an exploratory study, and the method has been described in detail elsewhere (Thomas et al., 2023). We approached families through social media platforms (e.g., Facebook) and illness-specific support organizations. Parents were eligible for the study if the following criteria were met: (1) their child was 0–12 years of age at the initial diagnosis of CHD, T1D, cancer, or asthma; (2) their child was currently under 18 years of age; (3) their child had received active/acute-care treatment for their condition in the last five years, and (4) they resided in Australia. We excluded parents of children with genetic or developmental conditions (e.g., ADHD, Autism) and parents of children with a pre-existing diagnosis of major depression and anxiety disorders.

### Measures

Parents completed an online survey that included items assessing family demographic characteristics, as well as measures of parent psychological distress (depression, anxiety, stress symptoms), children’s QoL, and items assessing USCN.

*Family demographics*. Respondents completed a series of questions assessing demographic characteristics, including age, relationship with the child, education, family status, and employment. Child’s characteristics included illness type, gender, current age, age at diagnosis, time since diagnosis, and treatment or management.

*Depression Anxiety and Stress Scales (DASS-21)* (Lovibond & Lovibond, 1995). This measure was used to assess the frequency of psychological distress on three subscales: depression (α = 0.93 in the current study), anxiety (α = 0.89 in the current study), and stress (α = 0.90 in the current study). Using a four-point Likert scale, parents were required to rate each of the 21 symptom-based items concerning their experience of each symptom over the past week (0 = “Did not apply to me at all” to 3 = “Applied to me very much”). Each of the three DASS-21 subscales consists of seven items, with scores calculated by summing items. As DASS-21 is the shorter version of the scale, the scores were multiplied by two as per scoring guidelines (Henry & Crawford, 2005). Higher scores indicate greater psychological distress, with well-established clinical cutoff values used to quantify the severity of parent psychological distress symptoms from “normal” to “severe.” Scores above 14, 10, and 19 indicate moderate to severe levels of depression, anxiety, and stress, respectively.

*PedsQL^TM^ 4 Generic Core Scale* (Varni et al., 2001). This parent-report scale is designed to measure caregivers’ perceptions of their child’s QoL and functioning during the past month. This scale comprises 23 items, which contribute to four subscales assessing different dimensions of children’s QoL: “physical functioning,” “emotional functioning,” “social functioning,” and “school functioning.” Parents rate each item using a five-point Likert scale that ranges from a score of 0 (“Never”) to 4 (“Almost Always”). For each subscale items were reverse scored, summed, and transformed to a 0–100 scale, such that higher scores represent better QoL. In addition to the four subscales, additional composite scores for “overall/total QoL” and “psychosocial functioning” are similarly calculated, with the latter composite measure combining items from the emotional, social, and school functioning subscales. Cronbach reliability alphas for all subscales and composite scores for the current study were high (all α over 0.84).

*USCN Survey*. As there is no universal instrument to assess unmet needs in families of children with different CHCs, we followed the approach used by Denham et al. (2020) for assessing unmet needs in carers of adults with mixed CHCs (Denham et al., 2020). Detailed information regarding the construction of this scale (including extraction of domain-level factor scores using exploratory principal component analysis) is provided elsewhere (Thomas et al., 2023). In brief, we developed a set of items drawing on surveys developed for cancer (Hodgkinson et al., 2007; Monterosso et al., 2006) and included 14 additional items derived from condition-specific literature. To ensure the suitability and acceptability of CHCs included in our study, we reviewed the wording of each item and modified any cancer-specific wording to ensure inclusivity. The USCN tool consists of 34 items (Thomas et al., 2023), which are used to derive scores that assess the level of unmet need across six domains: *care* needs (seven items, α = 0.87, e.g., managing your child’s health with the health care team), *physical and social* needs (seven items, α = 0.92, e.g., assisting your child in connecting with friends), *information* needs (four items, α = 0.88, e.g. having explanations given in a way that you can understand), *support* needs (nine items, α = 0.90, e.g., reducing any stress, you may be experiencing), financial needs (three items, α = 0.87, e.g., covering costs of medication for your child), and *child-related emotional* needs (four items, α = 0.87, e.g., worry about your child’s future). For each of the 34 items, parents rate their level of need for help over the past month using a four-point scale (i.e., 0: no need; 1: low need; 2: moderate need; 3: high need). For analysis, we counted the number of needs identified as “moderate” or “high” across items in each domain; then, we standardized the counts by dividing by the number of items in that domain and multiplying by 100. Domain scores could range from 0 to 100, with higher scores indicating a greater level of unmet need.

### Procedure

Prior to study commencement, ethical approval was obtained from the institutional review board (HEAG-H_53_2020) at Deakin University, Geelong, Australia. Participants were recruited by posting study flyers on social media platforms and organizational websites. After reading the background information, participants consent to complete the online survey using Qualtrics.

### Data Analysis

Descriptive statistics were used to characterize the demographic and illness-related characteristics of study participants, and group means, and standard deviations were calculated for the DASS-21, the PedsQL subscales, and the six USCN domain scales. ANCOVA examined differences in mean scores for DASS-21, PedsQL subscales, and USCN domains across CHCs (except asthma) with age at diagnosis and time since diagnosis included as covariates in these analyses. Post hoc analyses using Bonferroni correction for multiple comparisons examined the significance of differences across health conditions. Pearson correlations explored the strength and direction of associations between DASS-21, PedsQL subscales, and USCN domain scores with correlation values of ±0.1 representing a small effect, ±0.3 a medium effect, and ±0.5 a large effect (Cohen, 1977).

Hierarchical linear regression models were used to evaluate whether the indicators of parental psychological distress (i.e., DASS-21 depression, anxiety, and stress) were associated with (1) the child’s QoL domains and (2) the level of moderate to high USCN in the six USCN domains. Other child characteristics (illness type, age at diagnosis, time since diagnosis) and parent sociodemographic characteristics (age, education, employment, and marital status) were included as covariates in Step 1 of all regression models. Child QoL domains were entered in Step 2, and unmet need domains were entered in Step 3. The variance inflation factor was evaluated, revealing limited evidence for multicollinearity between variables. We used the change in *R*^2^ between each step to determine whether child QoL (PedsQL) and level of USCN accounted for significant additional variance in parent psychological distress outcomes. When significant associations were identified, we conducted a moderation analysis to explore whether the magnitude of the association varied as a function of child illness type. In these analyses, the significance of the interaction between CHCs and each significant variable from the regression analyses was tested, controlling only for diagnosis age and time since diagnosis, given that parental demographic factors did not reach statistical significance in regression analyses. Due to the small number of participants in the asthma group, we excluded data from these participants in moderation analysis. We used STATA v17 to perform hierarchical regression and moderation analysis (StataCorp., 2021) and SPSSv28 for all other analyses (IBMCorp., 2021). Post hoc power analyses indicated that with a sample size of 190, our hierarchical regression analyses have 80% power to detect a *f*^2^ of at least 0.09 (small to medium effect) change in *R*^2^ at each step with *p *<* *.05 (two-tailed). Post hoc power calculations for the full model (17 variables) indicated that with a sample size of 190 indicated, we had sufficient power to detect at least a medium effect size (*f*^2^* *=* *0.13) with 80% power and *p *<* *.05 (two-tailed).

## Results

Of the 300 participants who met the study eligibility criteria and accessed the survey, a total of 201 parents completed the online survey. After removing those parents who did not meet the eligibility criteria, the final study sample consisted of 194 parents (*M* age = 39 years, *SD *=* *6 years; range: 25–56 years), which represents a 65% response rate. The vast majority of respondents were mothers (93%), partnered (86%), and had at least an undergraduate degree (61%). Seventy-four percent of children were diagnosed with a CHC before five years of age. As shown in Table I, most children (80%) were under 10 years of age when the parent survey was completed. On average, parent surveys were completed at 3.3 years post-initial child diagnosis, although this varied across CHCs. All the data associated with asthma are provided as a Supplementary Data.

**Table I.** Demographic and Clinical Characteristics of Parents and Their Children With a CHC (N = 194)

	*n* (%)
Parent characteristics	
Age range^a^	
25–35 years	50 (26%)
36–45 years	109 (56%)
46–56 years	31 (16%)
Relationship with child	
Father	11 (6%)
Mother	180 (93%)
Education^a^	
Some college or less	72 (37%)
Undergraduate degree or greater	119 (61%)
Family status^a^	
Partnered	167 (86%)
Single/widowed	24 (12%)
Employment status^a^	
Employed	138 (71%)
Unemployed	52 (27%)
Children with CHCs	
Health condition	
CHD	97 (50%)
T1D	50 (26%)
Cancer	39 (20%)
Asthma	8 (4%)
Child sex	
Male	101 (52%)
Female	93 (48%)
Child current age	
≤5	76 (39%)
6–10	80 (41%)
11–16	38 (20%)
Child age at diagnosis	
≤5	144 (74%)
6–10	38 (20%)
11–16	12 (6%)
Time since diagnosis^b^	
Within one year	89 (46%) [CHD-20, T1D-47, Cancer-20, Asthma-2]
Within two years	22 (11%) [CHD-12, T1D-1, Cancer-9, Asthma-0]
Three or more years	83 (43%) [CHD-65, T1D-2, Cancer-10, Asthma-6]
Treatment/management received	
Surgery	115 (85%) [CHD-88, Cancer-27]
Insulin therapy	50 (100%) [T1D]
Chemotherapy	38 (97%) [Cancer]
Asthma plan	8 (100%) [Asthma]
Medications	54 (51%) [CHD-46, Asthma-8]

*Note*. CHD = congenital heart disease; T1D = type 1 diabetes mellitus.

a Missing values present.

b Frequency of time since diagnosis of individual conditions is given in brackets.

### Levels of Parent Psychological Distress and Children’s QoL

*DASS-21.* Group-level mean scores for parent psychological distress were as follows: depression: *M *=* *10.2, *SD *=* *10.7; anxiety: *M *=* *8.5, *SD *=* *9.4; and stress: *M *=* *17.0, *SD *=* *10.1 (Table II) suggesting that, at the level of overall group averages, parents in this study experienced “mild” symptoms of psychological distress across each of three DASS-21 subscales. However, when data were evaluated using clinical cutoff values for the DASS-21 subscales, we found that 40% of parents were experiencing moderate to severe stress, 38% moderate to severe anxiety, and 26% moderate to severe depression (Table II). There was a significant main effect of child CHC type on parent depression symptom severity (*F*(2, 181) =3.0, *p *<* *.05). Post hoc comparisons showed that compared to parents of children with CHD, mean levels of depression symptoms were significantly higher in parents of children with cancer. As shown in Table II, there was no significant main effect of CHC type on mean levels of parent anxiety and stress symptoms (*p *>* *.05).

**Table II.** Overall and Condition-specific Group Means (M) and Standard Deviations (SD) on Measures of Parents’ Psychological Distress, Children’s Quality of Life (QoL), and Level of Moderate to High Unmet Supportive Care Needs

**Psychological distress (DASS-21)** (range 0–42)	Total (*n* = 194)	CHD (*n* = 97)	T1D (*n* = 50)	Cancer (*n* = 39)	***F*(2, 181)** ^a^
Parent depression					
Mean (*SD*)	10.2 (10.7)	9.0 (9.6)^b^	11.2 (12.5)	12.9 (11.0)	3.0*
Moderate–severe threshold, *n* (%)	51 (26%)	23 (24%)	11 (22%)	16 (41%)	
Parent anxiety					
Mean (*SD*)	8.5 (9.4)	8.0 (9.0)	8.2 (9.9)	10.8 (10.2)	1.5
Moderate–severe threshold, *n* (%)	73 (38%)	36 (37%)	18 (36%)	18 (46%)	
Parent stress					
Mean (*SD*)	17.0 (10.1)	16.2 (9.9)	17.0 (10.5)	19.5 (10.4)	1.0
Moderate–severe threshold, *n* (%)	78 (40%)	35 (36%)	21 (42%)	21 (54%)	

Paediatric Quality of Life Inventory (PedsQL)—Parent Proxy Report (range 0–100)	Mean (*SD*)	Mean (*SD*)	Mean (*SD*)	Mean (*SD*)	

Total functioning	65.1 (21.0)	68.1 (22.0)^b^	67.9 (18.9)^b^	53.3 (18.8)	8.7**
Physical functioning	69.0 (25.9)	71.3 (26.6)^b^	78.1 (19.9)^b^	51.0 (24.5)	14.0**
Psychosocial functioning	63.0 (20.7)	66.4 (21.9)^b^	62.5 (19.7)	54.5 (17.9)	6.0**
Emotional functioning	56.8 (22.0)	60.5 (23.5)^b^	52.1 (21.4)	52.1 (18.2)	3.3*
Social functioning	70.1 (22.5)	71.1 (24.3)^b^	74.8 (20.3)	60.2 (18.8)	6.1**
School functioning	62.1 (25.6)	67.5 (25.1)^b^	60.8 (25.5)	51.2 (24.1)	6.5**

Moderate–high needs by domain^c^ (range 0–100)	Mean (*SD*)	Mean (*SD*)	Mean (*SD*)	Mean (*SD*)	

Care needs	37.1 (33.2)	27.5 (30.1)^b^	40.8 (34.2)	57.8 (29.3)	9.6**
Physical and social needs	35.2 (36.1)	24.8 (32.2)^b^	40.5 (37.0)	58.2 (35.5)	11.5**
Informational needs	29.7 (37.4)	22.6 (33.6)^b^	34.0 (39.0)	45.5 (39.6)	3.2*
Support needs	40.3 (33.6)	33.4 (31.2)^b^	45.1 (34.9)	56.6 (31.8)	5.2**
Financial needs	32.8 (39.0)	22.6 (34.5)	48.6 (43.2)^d^	40.1 (37.6)	4.5*
Child-related emotional needs	45.6 (39.2)	35.8 (37.3)^b^	46.5 (37.7)^b^	74.3 (32.6)	11.2**

*Note.* CHD = coronary heart disease; T1D = type one diabetes mellitus. Psychological distress (DASS-21): depression: normal 0–9; mild 10–13; moderate 14–20; severe 21–27; and extremely severe 28+; anxiety: normal 0–7; mild 8–9; moderate 10–14; severe 15–19; and extremely severe 20+; Stress: normal 0–14; mild 15–18; moderate 19–25; severe 26–33; and extremely severe 34+; Paediatric Quality of Life (PedsQL) parent proxy rated population norms (mean and SD): total functioning 82.29 (15.55), physical functioning 84.08 (19.70), psychosocial functioning 81.24 (15.34), emotional functioning 81.20 (16.40), social functioning 83.05 (19.66), school functioning 78.27 (19.64).

a ANCOVA test analyses excludes asthma group. Due to the small number of participants (n = 8), data for parents of children with asthma are not shown separately but are included in the total, and they are provided as Supplementary Data.

b Post hoc pairwise comparison between groups: significantly different from cancer; only borderline significance present between cancer and CHD for depression (p = .05).

c Moderate–high needs: Based on count of number of items indicated as a moderate to high unmet need. Higher scores indicate higher number of needs.

d Post hoc pairwise comparison between groups: significantly different from CHD.

* *p *<* *.05.

** *p *<* *.01.

*PedsQL.* When all children with CHCs were examined as a single group (*N *=* *194), mean parent proxy QoL scores were lowest for PedsQL emotional functioning (*M *=* *56.8, *SD *=* *22.0) and PedsQL school functioning (*M *=* *62.1, *SD *=* *25.6) (Table II). After controlling for age at diagnosis and time since diagnosis, there was a significant main effect of CHC type on all PedsQL subscales. For instance, for PedsQL total functioning (*F*(2, 181) = 8.7, *p *<* *.01), children with cancer had significantly poorer functioning than children with CHD and T1D.

### Levels of Moderate–High Unmet Needs among Parents of Children with CHCs

*Moderate–High Unmet Needs.* Table II displays the group means and standard deviations for each of the six unmet need domains*.* Moderate–high unmet need was highest in the child-related emotional need domain (*M *=* *45.6, *SD *=* *39.2), followed by needs in the support domain (*M *=* *40.3, *SD *=* *33.6) and care need domain (*M *=* *37.1, *SD* = 33.2). As shown in Table II, the average moderate–high needs in all domains differed significantly across the three main CHC types, such that higher group means were consistently documented in families of children with cancer followed by T1D. However, this same pattern did not emerge in the financial domain, such that financial needs were highest in the T1D group.

### Preliminary Bivariate Correlations among USCN, Children’s QoL (PedsQL) and Parent Psychological Distress (DASS-21)

There were statistically significant relationships between USCN domains, PedsQL, and DASS-21 subscales, with correlations reflecting medium to large effects (Table III). All USCN domains were positively associated with parents’ distress indicators, with the highest correlations found between unmet support needs and parent depression (*r *=* *0.60, *p *<* *.01), parent anxiety (*r *=* *0.60, *p *<* *.01), and parent stress (*r *=* *0.59, *p *<* *.01). All PedsQL subscales correlated negatively with the three indicators of parents’ psychological distress, such that higher child QoL scores (i.e., better child functioning) were associated with lower levels of parent psychological distress. The strongest correlations were between child QoL in the emotional functioning domain and parent symptoms of depression (*r *=* −*0.50, *p *<* *.01), anxiety (*r *=* −*0.50, *p *<* *.01), and stress (*r *=* −*0.50, *p *<* *.01). Each USCN domain was inversely related to all PedsQL functioning subscales, indicating that higher levels of unmet needs were consistently associated with poorer child functioning. For instance, a higher level of unmet physical and social needs (*r* = *−*0.67, *p *<* *.01) was associated with poorer child QoL in the school domain.

**Table III.** Pearson Correlations Among Parents’ Psychological Distress, Children’s Quality of Life (QoL), and Level of Moderate to High Unmet Supportive Care Needs in the Overall Sample (N = 194)

	DASS-21			PedsQL				Unmet supportive care need domains					
	1	2	3	4	5	6	7	8	9	10	11	12	13
**DASS-21**													
DASS-Depression (1)	1	0.81	0.78	−0.33	−0.50	−0.42	−0.33	0.53	0.46	0.50	0.60	0.45	0.48
DASS-Anxiety (2)		1	0.80	−0.28	−0.50	−0.40	−0.29	0.54	0.48	0.50	0.60	0.35	0.48
DASS-Stress (3)			1.00	−0.33	−0.50	−0.40	−0.33	0.53	0.43	0.47	0.59	0.30	0.50
**PedsQL**													
Physical functioning (4)				1.00	0.53	0.70	0.70	−0.44	−0.54	−0.31	−0.42	−0.21	−0.44
Emotional functioning (5)					1.00	0.64	0.63	−0.49	−0.60	−0.42	−0.51	−0.35	−0.47
Social functioning (6)						1.00	0.76	−0.46	−0.64	−0.36	−0.49	−0.32	−0.45
School functioning (7)							1.00	−0.51	−0.67	−0.37	−0.46	−0.30	−0.45
**Unmet supportive care need domains**													
Care needs (8)								1.00	0.67	0.65	0.74	0.52	0.63
Physical and social needs (9)									1.00	0.66	0.73	0.46	0.65
Informational needs (10)										1.00	0.74	0.50	0.65
Support needs (11)											1.00	0.55	0.71
Financial needs (12)												1.00	0.46
Child-related emotional needs (13)													1.00

*Note.* All the scores were statistically significant at.01 level; PedsQL = Paediatric Quality of Life.

### Evaluating the Contributions of Children’s QoL and USCN to Parents’ Psychological Distress

After adjustment for parent socio-demographic variables and child illness-related characteristics, child emotional functioning was the only QoL domain to have a significant association with each of parental depression, anxiety, and stress symptoms (Table IV). When USCN domains were included in the model at Step 3, higher unmet need in the support domain was significantly associated with greater parent depression symptoms (*B *=* *0.08, SE = 0.03, *p *<* *.05), higher anxiety (*B *=* *0.08, SE = 0.03, *p *<* *.01) and greater stress symptoms (*B *=* *0.09, SE = 0.03, *p *<* *.01). As shown in Table IV, we also found that higher unmet needs in the care domain were associated with higher parental stress symptoms (*B *=* *0.05, SE = 0.02, *p *<* *.05). Of note, all associations between children’s emotional functioning (QoL) and parent distress remained statistically significant after including the USCN measures in Step 3 of the regression models (depression: *B* = *−*0.15, SE = 0.03, *p *<* *.01; anxiety: *B* = *−*0.15, SE = 0.03, *p *<* *.01; stress: *B* = *−*0.17, SE = 0.03, *p *<* *.01).

**Table IV.** Stepwise Regression Models: Predicting Parent Distress Symptom Severity from Child Functioning (QoL) and Level of Moderate to High Unmet Supportive Care Needs in the Overall Sample (N = 194)

Variables	Depression			Anxiety			Stress		
	Model 1	Model 2	Model 3	Model 1	Model 2	Model 3	Model 1	Model 2	Model 3
	*B* (SE)	*B* (SE)	*B* (SE)	*B* (SE)	*B* (SE)	*B* (SE)	*B* (SE)	*B* (SE)	*B* (SE)
**Covariates**									
**Child factors**									
CHC–T1D	−0.15 (2.47)	0.58 (2.28)	0.69 (2.23)	−1.80 (2.20)	−2.41 (2.02)	−1.11 (1.94)	−2.04 (2.36)	−1.98 (2.12)	−0.30 (2.07)
CHC–CHD	−5.15 (2.43)*	−1.93 (2.14)	−0.33 (2.07)	−3.65 (2.18)	−1.39 (1.90)	0.63 (1.80)	−3.49 (2.33)	−0.29 (1.99)	1.54 (1.92)
CHC–asthma	−8.85 (4.20)*	−4.45 (3.63)	−1.75 (3.45)	−5.75 (3.76)	−2.09 (3.22)	1.49 (3.01)	−4.13 (4.02)	−0.16 (3.38)	3.25 (3.21)
Diagnosis age	−0.43 (0.34)	−0.54 (0.29)	−0.36 (0.28)	−0.41 (0.31)	−0.50 (0.26)	−0.40 (0.24)	−0.41 (0.33)	−0.52 (0.27)	−0.37 (0.26)
Time since diagnosis	−0.06 (0.29)	−0.43 (0.25)	−0.11 (0.25)	−0.26 (0.26)	−0.63 (0.22)**	−0.38 (0.21)	−0.56 (0.28)*	−0.95 (0.23)**	−0.62 (0.23)**
**Parent factors**									
University education (vs. less)	−2.81 (1.64)	−1.70 (1.41)	−0.45 (1.35)	−2.74 (1.47)	−1.89 (1.25)	−0.78 (1.17)	−2.42 (1.58)	−1.30 (1.31)	0.02 (1.25)
Single (vs. partnered)	0.67 (2.32)	−1.24 (1.99)	−1.95 (1.85)	2.37 (2.07)	0.70 (1.76)	0.43 (1.61)	1.24 (2.22)	−0.64 (1.85)	−0.67 (1.72)
Parents age (linear)	−0.24 (0.15)	−0.12 (0.13)	−0.04 (0.12)	−0.16 (0.13)	−0.05 (0.11)	0.01 (0.10)	−0.15 (0.14)	−0.05 (0.12)	0.00 (0.11)
Not working (vs. working)	−0.81 (1.79)	−0.64 (1.53)	−0.39 (1.44)	−2.30 (1.60)	−2.40 (1.36)	−2.13 (1.25)	−2.61 (1.71)	−2.60 (1.43)	−2.02 (1.34)
**PedsQL**									
Physical functioning		−0.00 (0.04)	−0.00 (0.03)		0.04 (0.03)	0.04 (0.03)		−0.01 (0.03)	0.01 (0.03)
Emotional functioning		−0.21 (0.04)**	−0.15 (0.03)**		−0.22 (0.03)**	−0.15 (0.03)**		−0.23 (0.03)**	−0.17 (0.03)**
Social functioning		−0.09 (0.05)	−0.05 (0.04)		−0.09 (0.04)*	−0.06 (0.04)		−0.05 (0.04)	−0.03 (0.04)
School functioning		0.03 (0.04)	0.06 (0.04)		0.03 (0.04)	**0.07 (0.03)** *		−0.00 (0.04)	0.02 (0.04)
**Unmet supportive care need domains**									
Care needs			0.04 (0.03)			0.04 (0.02)			**0.05 (0.02)** *
Physical and social needs			−0.01 (0.03)			0.01 (0.02)			−0.02 (0.03)
Informational needs			0.01 (0.02)			0.01 (0.02)			0.00 (0.02)
Support needs			**0.08 (0.03)** *			**0.08 (0.03)** **			**0.09 (0.03)** **
Financial needs			0.03 (0.01)			−0.00 (0.01)			−0.02 (0.01)
Child-related emotional needs			−0.00 (0.02)			0.00 (0.02)			0.02 (0.02)
*F*	1.75	7.34**	7.88**	1.82	7.82**	8.84**	2.02*	8.86**	9.16**
Adj *R*^2^	0.03	0.30	0.41	0.03	0.32	0.44	0.04	0.35	0.45
*R* ^2^ change		0.27**	0.11**		0.28**	0.13**		0.30**	0.11**

*Note. B* = beta coefficients; SE = standard errors; PedsQL = pediatric quality of life; CHC = chronic health conditions; CHD = congenital heart disease; T1D = type one diabetes. Models 2 and 3 contained covariates of chronic health condition, diagnosis age, time since diagnosis, parent’s education, parent’s marital status, parent’s age, parent’s employment (entered in Model 1). Significant associations are in bold.

* *p *<* *.05.

** *p *<* *.01.

Moderation analyses revealed that the associations between these variables did not vary by child illness type, indicating that associations were consistent across health conditions.

## Discussion

This study aimed to assess anxiety, depression, and stress in parents of children with a CHC and to explore the relative contribution of unmet needs and child’s QoL to parental distress. On average, parents in our study exhibited mild levels of distress, and approximately 26–40% of parents were found to have moderate or higher levels of depression, anxiety, or stress. While limitations in our sample’s representativeness need to be acknowledged, these findings align with previous research that has shown elevated levels of psychological distress in parents of children with cancer, T1D, and CHD (Chen et al., 2023; Cousino & Hazen, 2013; Jantien Vrijmoet-Wiersma et al., 2008; Kolaitis et al., 2017; Whittemore et al., 2012). The current study’s exploration of the relative contribution of child QoL domains and USCN domains to parent distress is novel and adds to the field. Our multivariable analyses suggested that the child’s emotional functioning and unmet needs in the support domain were key contributors to all three distress indicators. While the cross-sectional nature of our study suggests caution is needed when interpreting the direction of associations presented in our results, our findings suggest that the additional work needed to confirm both the associations and the direction of effect is warranted.

### Psychological Distress among Parents and Children’s QoL

Despite limitations to the way our sample was recruited, the proportions with elevated depression and anxiety seen in our study are similar to findings reported in the literature. For instance, similar to our findings for parents of T1D children, a recent systematic review and meta-analysis estimated that around 22% of parents of children with T1D experienced elevated levels of depression (Chen et al., 2023). Similarly, a recent large study assessing the long-term psychological impact of caring for a child with a range of heart conditions found that 42% of mothers experienced elevated anxiety (Wray et al., 2018), a level similar to estimates found in the current study. However, rates of depression were lower in that study than in the current study (12% vs. 24%), which may reflect the older age of children (average 12–13 years) in that study. While for parents of children with CHD, the period of diagnosis and initial treatment is most distressing (Nayeri et al., 2021), the long-term impact of caring for a child with CHD for parents is still unclear with not all work showing elevated levels of depression, anxiety, or somatization continue over time (Kolaitis et al., 2017). Our results, coupled with findings from previous literature, suggest the need to continue investigating possible psychosocial interventions to improve coping and reduce distress for parents embarking on long-term care of their child with a CHC.

In keeping with previous reports that children with CHCs are vulnerable to experiencing long-term reductions in health-related QoL (Drakouli et al., 2015; Klassen et al., 2011), the group-level means on child QoL measures (PedsQL Parent-Report) in our study suggested that children in our sample had significantly worse QoL in comparison to population-level norms (Varni et al., 2003). Interestingly, the mean PedsQL scores in our sample of children were also lower than those reported in a US population-based study involving PedsQL parent ratings for children with cancer, CHD, T1D, and asthma (Varni et al., 2007). However, despite our lower scores, the pattern of results for the QoL domains in our study is consistent with findings from this US study (Varni et al., 2007), which also found that emotional and school functioning were the most significantly impacted QoL domains in children with CHD, T1D, cancer, and asthma regardless of whether parent proxy or child reports of QoL were used (Varni et al., 2007). It is possible that our convenience sampling methods (e.g., online support groups and charity organizations) increased the likelihood of recruitment bias, as other work has shown that online recruitment is more likely to attract families with higher support needs (Kadravello et al., 2021). If our findings are confirmed in larger studies, interventions to enhance pediatric emotional and school functioning across CHCs may be warranted. As children in our sample were, on average, three years post-diagnosis, our results, if confirmed, may suggest that interventions may also be needed in the *“chronic long haul”* phase of these conditions (Rolland, 1987).

### Factors Associated with Parents’ Psychological Distress

Our finding regarding the role that unmet support needs can play in parents’ distress is consistent with the results of a recent longitudinal study that examined the mental health-related QoL in mothers of children with CHD (Ehrler et al., 2023). That study found that approximately 25% of these mothers experienced consistently low mental health QoL over time and that a lack of social support was a risk factor for belonging to this group (Ehrler et al., 2023). Another systematic review (*N* = 25 studies) of studies of families of children with CHD concluded that parents with fewer psychosocial resources and lower levels of support may be at risk of experiencing higher psychological distress and lower well-being over time (Jackson et al., 2015). Based on these findings, addressing unmet support needs in families of children with CHCs may be one strategy to alleviate distress and enhance the well-being of parents.

A systematic review synthesizing qualitative evidence of the impact of T1D on parents (*N* = 14 studies) found its complexities and unpredictable nature made it a “*very tiring disease*” for parents, causing constant worry, a need for vigilance, and increased caring responsibilities (Kimbell et al., 2021). The review also emphasized that having appropriate support makes parents feel less isolated, enabling them to vent their frustrations and that a lack of professional and informal support creates anxiety in parents (Kimbell et al., 2021). Our finding that a greater number of unmet support needs is associated with elevated distress aligns with this latter finding. These results are similar to the adult cancer carer literature, with a systematic review (*n* = 29 studies) showing that higher unmet needs are associated with elevated carer psychological distress (Lambert et al., 2012), with other work showing that the influence of the different domains varies by psychosocial factor studied (Cheng et al., 2022). For instance, unmet personal and emotional needs were associated with anxiety and depression in carers of adolescents and young adults with cancer (Cheng et al., 2022), while unmet communication and family needs were associated with anxiety in carers of adults with cancer (Sklenarova et al., 2015). Items in the “support” domain reflect a mix of needs relating to parents managing their own stress levels, managing the feelings of other children they may have and finding support services their child could use. The range of items in this domain may contribute to its association with the three distress indicators. While our findings suggest that more support in this domain might assist parents in managing feelings of psychological distress, more work is needed utilizing longitudinal study designs to confirm these findings.

Few studies to date have explored unmet needs in families of children with various CHCs (Thomas et al., 2023). Moreover, few studies have examined links between unmet needs and parental distress in this population, with most work in this area to date focusing on parents of children with cancer (Aziza et al., 2019; Wang et al., 2022). The current study examined these relationships across a range of common childhood CHCs and explored how unmet needs and child QoL directly influence parent distress levels. However, due to the inherent limitations of cross-sectional designs employed by our own research and the study conducted by Wang et al. (2022) and Aziza et al. (2019), it is not possible to draw causal inferences about whether child functioning influences parent functioning or vice versa in families of children with CHCs. Longitudinal studies are needed to address such limitations and to evaluate the directionality of the observed effects.

Poor child QoL in the emotional domain was linked with all three measures of parental distress: depression, anxiety, and stress. Given that emotional adjustment difficulties are common in children with CHCs (Barlow & Ellard, 2006), these findings might suggest that interventions for children’s emotional distress may also positively impact parent’s mental health and well-being. However, given the study’s cross-sectional nature, it is also possible that parent distress leads to reduced QoL in children as suggested in previous research in pediatric cancer and other areas (Bakula et al., 2019, 2020). Accordingly, larger longitudinal studies are required to replicate our study findings and clarify the direction of the cross-sectional associations documented in our work.

### Strengths and Limitations

To our knowledge, this is one of the few studies to examine associations between child QoL, unmet support needs and psychological distress in parents of children living with various CHCs. However, there are some limitations that need to be acknowledged. First, we recruited during the Coronavirus disease-2019 pandemic with strict lockdowns in place in much of Australia during this period. As such, we cannot rule out the possibility that the pandemic had a potential impact on parents’ responses. Second, we invited parents into the study through online support groups and charity organizations, which could limit the generalizability of the findings as parents joining these online support groups may be seeking support and assistance. This cross-sectional survey utilized convenience sampling, with the majority of respondents being mothers, who completed all measures of interest. While this approach may overestimate the magnitude of the associations between variables, it is an approach routinely used in studies of childhood CHCs (Cousino & Hazen, 2013). The proportion of participants with a tertiary education is perhaps higher than expected for the broader Australian population, which may partially limit the generalizability of the study findings.

To help address the potential impact of time post-diagnosis on the observed pattern of findings, all analyses involved adjustment for this covariate. However, given we were unable to adjust for the level of unmet medical needs, we cannot rule out the potential role of this variable in influencing the observed pattern of findings. Furthermore, small sample sizes for the different CHC groups may have limited the statistical power of our moderation analyses. While our sample may under-represent families of children with different conditions, the relative numbers of each health condition in our sample roughly reflect the incidence of these conditions in Australian children, with the exception of asthma (AIHW, 2019, 2023; Youlden et al., 2020). Other significant limitations include that we did not require participants to disclose their ethnic origin and we excluded children with genetic, developmental, or emotional disorders. Consequently, investigating ethnic or cultural disparities in unmet needs and exploring unmet needs and QoL of children with developmental or emotional disorders are important areas for future research. While these limitations are important when interpreting the findings, the associations we found among the variables we explored suggest a relationship. To further advance this field, more work is needed to investigate the direction and strength of associations between different variables using larger samples and longitudinal designs that include children’s own assessment of their QoL. These types of studies will overcome the challenges posed by cross-sectional data and establish more robust causal relationships.

### Conclusion

Our cross-sectional study shows that elevated psychological distress is experienced by a large proportion of parents in our sample. Greater parental distress was associated with lower child emotional functioning (QoL), as well as higher unmet needs in the support domain. Notably, these associations were not moderated by child illness type, suggesting that these factors may be relevant for children and families living with childhood chronic illness, irrespective of their specific diagnosis. Except for time-since-diagnosis (which was adjusted for in all analyses), parent distress was not significantly associated with other child and parent socio-demographic factors. These findings likely have implications for family-centered assessment and intervention, including the provision of routine evaluation and referrals to address parents’ psychological distress and USCN. Similarly, it is likely that the provision of timely, evidence-based psychological interventions for these children will help improve their emotional functioning and may also have secondary benefits in helping to alleviate psychological distress experienced by their parents and/or families.

Given that this study represents an initial exploratory investigation in a relatively under-researched area, larger longitudinal studies are needed to replicate findings in diverse cultural and ethnic groups, as well as evaluate possible bidirectional relationships between child functioning (QoL), USCN and parent psychological distress in families of children living with CHCs.

## Ethical Approval

Low risk ethical approval from Deakin University (HEAG-H_53-2020). Informed consent to participate in the study has been obtained from all the participants.

## Supplementary Material

jsad074_Supplementary_Data

## Data Availability

Due to ethical and privacy reasons data are not publicly available. Datasets generated during the current study will be accessible from the corresponding author upon reasonable request.
