# Supplementary material for: Psychological Distress Among Parents of Children With Chronic Health Conditions and Its Association With Unmet Supportive Care Needs and Children’s Quality of Life
Source: J Pediatr Psychol. 2023 Oct 14;49(1):45–55. doi: 10.1093/jpepsy/jsad074 (PMC10799716; doi:10.1093/jpepsy/jsad074)
Supplement: jsad074_Supplementary_Data [file jsad074_supplementary_data.docx]

**Supplementary file**

**Data generated from asthma group**

Table 1

*Descriptive statistics of parent’s psychological distress, parent reported child’s quality of life (QoL), and level of moderate to high unmet supportive care needs for asthma (n=8)*

| **Psychological distress (DASS-21)**  (Range 0-42) |  |
| --- | --- |
| **Parent Depression** |  |
| Mean *(SD)* | 4.2 (5.8) |
| Moderate-severe threshold, *n* (%) | 1 (13%) |
| **Parent Anxiety** |  |
| Mean *(SD)* | 4.7 (6.4) |
| Moderate-severe threshold, *n* (%) | 1 (13%) |
| **Parent Stress** |  |
| Mean *(SD)* | 14.5 (8.1) |
| Moderate-severe threshold, *n* (%) | 1 (13%) |
| **Paediatric Quality of Life Inventory**  **(PedsQL)-Parent Proxy Report**  (Range 0-100) | **Mean (SD)** |
| Total Functioning | 68.2 (11.6) |
| Physical functioning | 71.0 (14.5) |
| Psychosocial Functioning | 66.6 (14.8) |
| Emotional Functioning | 65.0 (16.2) |
| Social Functioning | 77.5 (15.8) |
| School Functioning | 57.5 (24.2) |
| **Moderate-high Unmet needs ^††^** (Range 0-100) | **Mean (SD)** |
| Care needs | 28.5 (34.1) |
| Physical and social needs | 16.0 (19.3) |
| Informational needs | 12.5 (35.3) |
| Support needs | 15.2 (27.1) |
| Financial needs | 20.8 (35.3) |
| Child-related emotional needs | 18.7 (29.1) |

**Note**: **Psychological distress (DASS-21):** **Depression:** Normal 0-9, Mild 10-13, Moderate 14-20, Severe 21-27, and extremely Severe 28+; **Anxiety:** Normal 0-7, Mild 8-9, Moderate 10-14, Severe 15-19, and extremely Severe 20+; **Stress:** Normal 0-14, Mild 15-18, Moderate 19-25, Severe 26-33, and extremely Severe 34+**; Paediatric Quality of life (PedsQL)** parent proxy rated population norms (Mean and SD): Total functioning 82.29 (15.55), Physical functioning 84.08 (19.70), Psychosocial functioning 81.24 (15.34), Emotional functioning 81.20 (16.40), Social functioning 83.05 (19.66), School functioning 78.27 (19.64). **Moderate-high needs: ^††^** Based on count of number of items indicated as a moderate to high unmet need. Higher scores indicate higher number of needs.
